# Assessing the Use of Influenza Forecasts and Epidemiological Modeling in Public Health Decision Making in the United States

**DOI:** 10.1038/s41598-018-30378-w

**Published:** 2018-08-17

**Authors:** Colin Doms, Sarah C. Kramer, Jeffrey Shaman

**Affiliations:** 1 Quantitative Methods in the Social Sciences, Graduate School of Arts and Sciences, Columbia University, 109 Low Memorial Library, MC 4306, 535 W 116th Street, New York, New York 10027 United States of America; 2 Department of Environmental Health Sciences, Mailman School of Public Health, Columbia University, 722 W 168th Street, New York, New York 10032 United States of America

## Abstract

Although forecasts and other mathematical models have the potential to play an important role in mitigating the impact of infectious disease outbreaks, the extent to which these tools are used in public health decision making in the United States is unclear. Throughout 2015, we invited public health practitioners belonging to three national public health organizations to complete a cross-sectional survey containing questions on model awareness, model use, and communication with modelers. Of 39 respondents, 46.15% used models in their work, and 20.51% reported direct communication with those who create models. Over half (64.10%) were aware that influenza forecasts exist. The need for improved communication between practitioners and modelers was overwhelmingly endorsed, with over 50% of participants indicating the need for models more relevant to public health questions, increased frequency of telecommunication, and more plain language in discussing models. Model use for public health decision making must be improved if models are to reach their full potential as public health tools. Increased quality and frequency of communication between practitioners and modelers could be particularly useful in achieving this goal. It is important that improvements be made now, rather than waiting for the next public health crisis to occur.

## Introduction

Numerical forecasting—the computational real-time generation of calibrated predictions on time scales allowing application and validation—has a long history of use in the fields of weather and climate^1–3^. In recent decades, numerical forecasts have been developed for and applied to a number of new industries and disciplines, including agriculture^4,5^, air quality^6,7^, consumer activity^8–10^, fiscal policy^11^, and political elections^12^. These forecasts allow stakeholders to prepare for predicted future events and to respond accordingly. For example, forecasts of crop yields help governments decide whether food must be imported to meet population needs, and inform decisions concerning the receipt of emergency food aid^5^. Meanwhile, many companies use sales forecasting when deciding how much of a product to stock in order to maximize profits^8^. In public health, forecasting methods have been developed using mathematical models and Bayesian inference methods and used to predict the growth and spread of infectious diseases such as influenza^13–18^, dengue^19–21^, Ebola^22–24^, and, most recently, Zika^25,26^.

In the United States, influenza is estimated to kill tens of thousands of people and cost over $87 billion each year^27^. Several research groups, including ours, have developed forecasts of influenza incidence in the United States^28^. These forecasts estimate future incidence levels for a developing influenza outbreak with particular focus on metrics such as when the outbreak will be most severe or how many cases will occur during the most severe week of the outbreak. In our own efforts, forecasts have been generated for municipalities and states throughout the US, as well as for several European countries, and operationalized for real-time delivery over an online portal^29^. These quantitative forecasts are updated weekly during the flu season and have the potential to reduce morbidity, mortality, and healthcare spending by influencing decision making and resource allocation among healthcare providers, public health practitioners, and the general public alike. For example, hospitals may use the forecast peak timing of an influenza outbreak to prepare for an influx of patients, and the public may be more motivated to practice proper hand hygiene when high influenza incidence is predicted. However, these benefits will only be fully realized if public health practitioners are aware of this work and use these findings in decision making.

Research on the extent to which public health practitioners utilize mathematical models is limited. Indeed, to our knowledge, no existing studies assess the use of mathematical models in public health decision making in the US. Driedger *et al*.^30^ interviewed four public health practitioners and four mathematical modelers in order to assess the integration of modeling in decision making during the 2009 influenza pandemic in Canada. They concluded that improved communication between practitioners and modelers was needed. Specifically, they found that practitioners desired greater clarity in model interpretation, and modelers wanted a better understanding of the questions practitioners needed modeled. Both groups expressed the need for longstanding partnerships in order to increase efficiency, understanding, and trust between the two groups. An earlier Canadian study also found need for more and better communication between practitioners and modelers^31^. Most recently, Moss *et al*. shared weekly forecasts of influenza activity in Melbourne, Australia with the local health department, and updated their forecasts based on insights from the practitioners there. They report that these collaborations were instrumental in improving forecast accuracy^32^.

Here, we addressed these issues using a different approach. We employed a short survey to assess the extent to which US public health professionals are aware of and use mathematical models, including influenza forecasts, in making decisions on the job. Through this preliminary effort, we seek to build the evidence base describing the integration of numerical epidemiological modeling, including seasonal influenza forecast, and public health decision making.

## Methods

### Participants

We recruited survey participants via email through contacts at three U.S. public health organizations: the Association of State and Territorial Health Officials (ASTHO), the Council of State and Territorial Epidemiologists (CSTE), and the National Association of County and City Health Officials (NACCHO). Although we do not know how many practitioners ultimately received a link to our survey, these organizations represent a large number of employees in the fields of public health, epidemiology, and influenza control across the US, ensuring that our survey was sent to a representative sample of US public health practitioners.

### Materials

We designed a survey containing 25 multiple-choice and Likert scale questions (see Supplementary Information). The survey included questions on basic demographics, awareness of influenza forecasts, whether the respondents used epidemiological models in their work, and whether they applied model results to public health decision making. Participants were also asked if they communicated with modelers, and how such communication could be improved. Finally, we inquired about personal use of influenza vaccination for the current and previous seasons. This work was approved by and performed under Columbia University Medical Center IRB (approval number CUMC IRB-AAAO9952). The IRB-approved survey was distributed online through SurveyMonkey, and informed consent was acquired through a checkbox on the survey’s first page. All results were de-identified.

### Procedure

Participants were recruited through broadcast emails to the members of each of the three organizations. We collected responses over roughly a six-month period. Most of the responses from one organization were collected during March and April 2015, and other responses were completed during September 2015. The difference in timing was due to differing availability to contact their members. In addition, in August 2015 we changed the word ‘survey’ to ‘assessment’ in order to comply with a request from one organization and gather more responses. Thus, a majority of participants saw ‘assessment’, although we believe this wording change had little effect, if any, on the results.

## Results

### Data

A total of 51 individuals responded to the survey, 42 (82.4%) of whom indicated employment in a public health field. Because we are primarily interested in awareness and use of models among public health practitioners, we restricted our analysis to these individuals. Furthermore, we removed three other participants whose responses were inconsistent; specifically, two individuals reported a frequency of model use while simultaneously reporting that they did not use models in their work, and one participant reported acquiring influenza data from both Columbia University and none of the sources listed on the survey. This left us with data on 39 participants. All 39 participants reported that their work-related responsibilities included planning for and dealing with influenza outbreaks. The majority of respondents (38, 97.4%) worked for the government, and one worked for an NGO.

### Demographics

Demographic information is summarized in Table 1. Briefly, the majority of respondents (22, 56.4%) were between the ages of 30 and 49. Years in public health was fairly evenly distributed, with the largest group being those who had been in the field for 4–6 years (13, 33.3%). Two-thirds of respondents (26, 66.7%) reported being female, and most (33, 84.6%) had at least a graduate degree. Respondents were spread geographically across 35 states and territories. Regional totals are based on divisions defined by the United States Census Bureau^33^. Due to the small sample size obtained here, it was not plausible to use more narrow regional divisions.

**Table 1:** Demographic Characteristics of 39 Public Health Practitioners Surveyed Concerning Awareness and Use of Mathematical Models.

Gender	
Female	26 (66.7%)
Male	12 (30.8%)
Age	
18–29	9 (23.1%)
30–49	22 (56.4%)
50–64	5 (12.8%)
65+	1 (2.6%)
Degree obtained	
Bachelor’s degree	6 (15.4%)
Graduate degree	33 (84.6%)
Years in public health	
0–3 years	5 (12.8%)
4–6 years	13 (33.3%)
7–10 years	6 (15.4%)
11–15 years	8 (20.5%)
16+ years	6 (15.4%)
Region	
West	10 (25.6%)
South	10 (25.6%)
Northeast	7 (17.9%)
Midwest	10 (25.6%)
Territories	1 (2.6%)

### Use of Models

**Figure 1:**
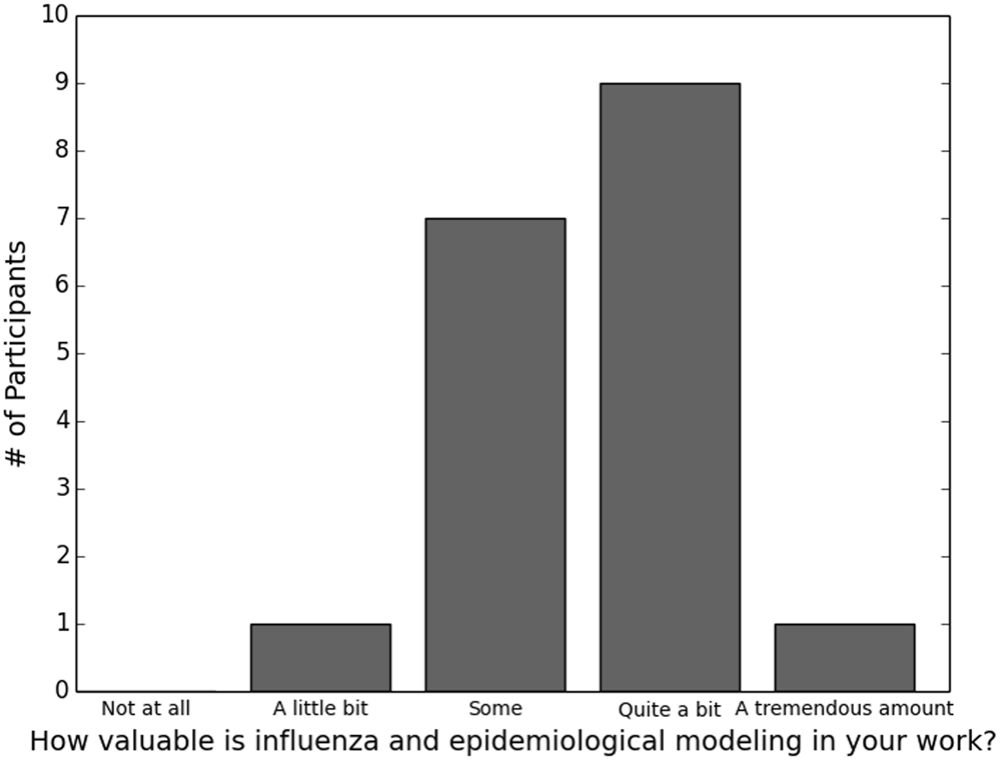
Reported value of models among eighteen public health practitioners who reported using models on the job.

**Figure 2:**
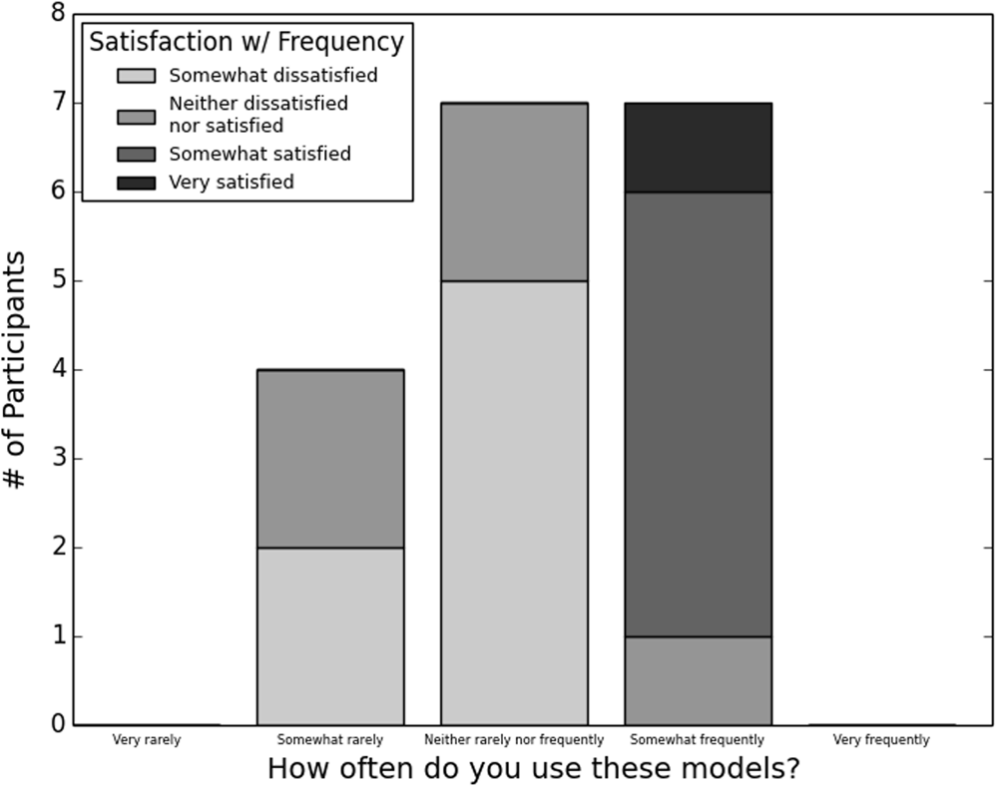
Reported frequency of model use and satisfaction with this frequency among eighteen public health practitioners who reported using models on the job.

Almost half of respondents (18, 46.2%) reported using models in their work, and that use differed significantly by region (two-tailed Fisher’s exact test, *P* = 0.0311; regions are defined as described under “Demographics” above). Specifically, use was highest in the West and lowest in the South and Midwest. Use of models was not significantly related to other demographic variables. Most of these individuals considered the models to be valuable (Fig. 1) and used them relatively frequently (Fig. 2). Satisfaction with this frequency varied (Fig. 2), but was significantly higher with higher frequency of use (two-tailed Fisher’s exact test, *P* = 0.003).

### Communication with Modelers

**Figure 3:**
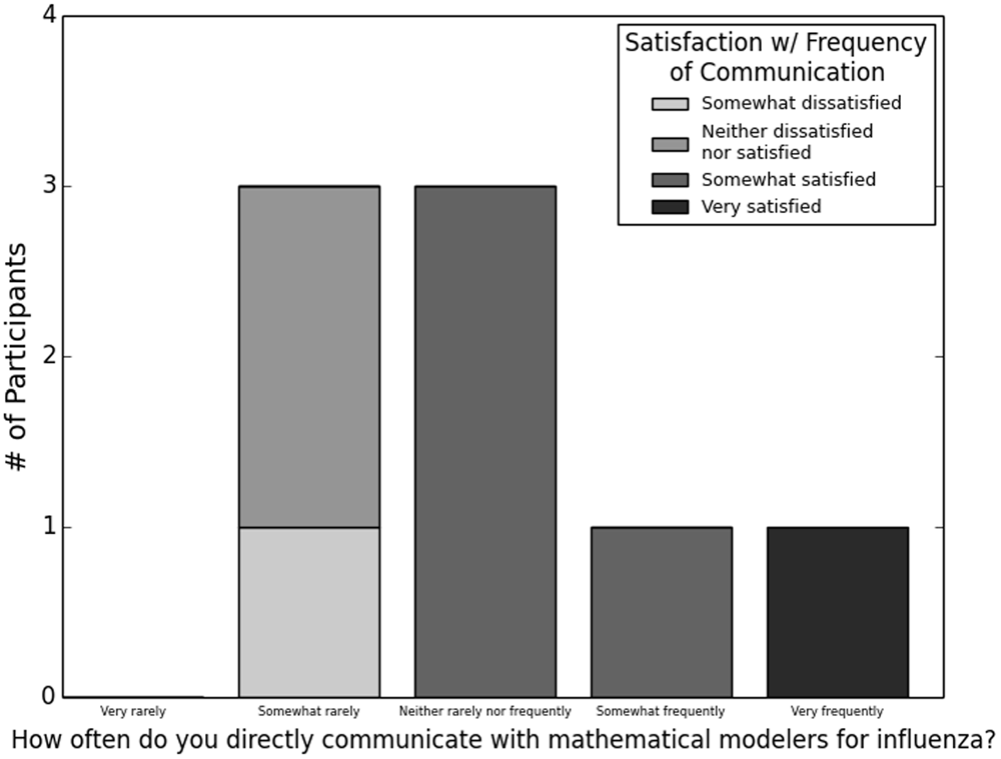
Frequency with which public health practitioners communicated with people who develop mathematical models of influenza and satisfaction with this frequency among eight participants who reported ever communicating.

A total of eight (20.5%) respondents indicated communication directly with those who develop and create models; seven of these individuals also used models in their own work. Although this interaction occurred fairly rarely (Fig. 3), most participants were satisfied with this low level of communication. Again, there was a tendency for satisfaction to be higher with more frequent communication (two-tailed Fisher’s exact test, *P* = 0.043), but the sample size (n = 8) was very small.

When asked how communication with modelers could be improved, 26 (66.7%) respondents indicated that models should be more relevant to public health questions, 23 (59%) wanted increased frequency of telecommunication, 20 (51.3%) desired more plain language from modelers, and 13 (33.3%) wanted more face-to-face conversation. Three individuals entered their own responses, which were: “Models designed taking into account US jurisdictions outside the contine[n]tal US”, “Provide more information on the value of models to support questions from other health professionals and the media”, and “Greater availability of models. I did not know these existed”.

### Awareness of Influenza Forecasts

Twenty-five (64.1%) respondents were aware that forecasts for influenza are available, and 18 (72%) of these individuals had seen one in the past 12 months. These rates were no higher among those who used models in their work than among those who did not (chi-squared test, *P* = 1 and *P* = 0.4423, respectively). Furthermore, participant ratings of model usefulness did not differ significantly based on whether or not the participant was aware of or had seen forecasts (Fishers exact test, *P* = 0.509 and *P* = 0.597, respectively).

Only seven participants (18% of the total; 38.9% of those who had seen a forecast) reported that they or their colleagues had accessed Columbia University’s forecasts specifically. Among these seven, three agreed that the forecasts were trustworthy and the other four rated their trustworthiness as neutral. Most (five) said that the forecasts were released neither frequently nor infrequently, and “somewhat frequently” and “very rarely” were also endorsed by one individual each. Finally, only two respondents actually used these forecasts in decision making, with one reporting that the forecasts changed communication strategies with the public and stakeholders and influenced preparedness in a healthcare facility, and the other reporting that the forecasts “supported our regional risk activity assessment”.

### Data Sources

Of the 39 respondents, 34 (87.2%) reported obtaining influenza incidence or forecast data from the Centers for Disease Control and Prevention (CDC), 14 (35.9%) from Google Flu Trends, five (12.8%) from Columbia University, four (10.3%) from HealthMap FluCast, and seven (18%) from other sources, most commonly state and local ILI (influenza-like illness) reports. Only one respondent reported using no sources at all. However, we note that most (13/14) respondents who said they used Google Flu Trends did so after Google Flu Trends was taken offline in July 2015. Thus, although these individuals used Google Flu Trends in the past, we do not know if they continued to access influenza data from other sources.

## Discussion

Despite the potential benefits of using mathematical models, including forecasts, to address public health questions, knowledge of whether and how US-based public health practitioners incorporate model-generated information into decision making is limited. Here, we examine this situation using a cross-sectional survey of 39 public health practitioners in the United States.

Almost half of respondents reported using models in some capacity, and most rated the value of models highly. Future work should determine why some participants view models as more valuable or use models more frequently than others, and efforts should be taken to increase access to, utility of, and user-friendliness of models and model-generated information. Influenza forecasts in particular could be of use to public health practitioners, in that accurate predictions of influenza outbreak metrics, such as peak timing and intensity, could inform vaccination strategies, resource allocation, and communication with the public. Notably, all of our respondents reported that they frequently work with influenza outbreaks. For this reason, it was promising to observe that almost half of surveyed practitioners had seen or had a colleague who had seen an influenza forecast in the past 12 months. However, those who had seen a forecast were not more likely to use models in their work than those who had not, suggesting that these forecasts are not often put to practical use. In fact, although we only asked about forecast use among those who accessed Columbia’s forecasts specifically, only two of seven respondents reported actually using the forecasts in public health decision making.

Suboptimal use of available forecasts is an issue in many fields, and is particularly well-studied in agriculture. A study of the use of monsoon forecasts in India found that many farmers complained that forecasts were not available when they were needed, emphasizing the importance of generating forecasts with appropriate lead time^34^. Additionally, a separate review of forecast use in agriculture implicates insufficient forecast quality, both real and perceived, for preventing forecast use in decision making^35^. Due to the potential severity of influenza, it is logical that the prospect of acting on an inaccurate forecast is concerning to practitioners. Kusunose and Mahmood^35^ suggest that expectations of forecasts might be made more realistic by incorporating the degree of uncertainty associated with predictions, something our group has developed for influenza^15,36^. Future studies should further explore the reasons public health practitioners are hesitant to rely on influenza forecasts, as well as the formats and modes of delivery most useful to practitioner work, so that such concerns can be better addressed.

Perhaps our most salient result concerns the overwhelming endorsement of several ways for improving public health practitioner communication with modelers. This finding is in line with previous reviews and qualitative studies^30–32,37,38^. To improve communication between modelers and practitioners, both knowledge- and trust-related issues that prevent practitioners from using models effectively should be addressed. For instance, the development of specific guidelines on using mathematical models to answer public health questions may help to clear up misconceptions concerning the capacity of models. Additionally, past qualitative work has found consistency of language and clear communication of model assumptions to be of particular importance^30,37,38^. Increased trust in modeling methods and results might also be cultivated by forming longstanding collaborations between practitioners and modelers^31,32^. Future work could survey practitioners participating in collaborations with modelers to determine which communication practices have been most and least effective. While nuanced and detailed communication efforts will be necessary, basic informational campaigns can also play a role: One participant did not know that models existed before taking our survey.

Finally, in addition to the questions posed concerning communication frequency and quality, future surveys should assess how participants communicate with modelers, what topics are discussed, and their endorsement of a variety of ways to improve communication. They should also allow for qualitative responses from participants; these responses could suggest effective methods for increasing communication quality and frequency that may be less obvious to modelers.

### Limitations

Despite the novelty of this work, several limitations should be addressed. First, although we attempted to contact a large number of public health practitioners, our response rate was small, making it difficult to draw concrete conclusions, or to statistically assess whether model use differed by variables such as years working in public health. Furthermore, our sample is a convenience sample, and may not be representative of the wider group of public health practitioners. Unfortunately, we know neither the demographic distributions among nonrespondents nor the number of practitioners our survey reached, and can therefore report neither adjusted results nor an overall response rate. However, given that our sample is likely biased toward practitioners with greater knowledge of and interest in mathematical models, we expect that these measures would be even lower among a truly random sample. Thus, our conclusion that model use is below 50%, at least, is likely to hold among US-based public health practitioners in general.

We also note that the definition of the word “model” in our survey was ambiguous. Although we hope that the questions on influenza forecasts prompted participants to think in terms of mechanistic models, it is possible that some respondents took the survey with other types of models, such as regression models, in mind. Similarly, exactly what constitutes model “use” could be anything from simply viewing model output to being actively involved in the development and execution of a model; unfortunately, we cannot tell where each participant falls on this spectrum.

Given that three data points were removed due to inconsistent responses, and that several participants reported using an unavailable data source (GFT), an increased focus on response credibility is indicated. A clear definition of “model” and “model use” will be instrumental in increasing the credibility of future survey results. Reliability can be further enhanced by asking respondents to elaborate their responses, such as through providing a specific categorization or description of the context and form of model used.

## Conclusions

Among 39 surveyed public health professionals, both model use and familiarity with influenza forecasts were reported by almost half of participants, but communication with model developers was rare. Improved communication between modelers and practitioners in particular seems to be key for increasing the frequency and effectiveness of model use among public health practitioners. Although more research on why forecasts and other models are not commonly used is necessary, initial improvements should be made now, in the absence of urgent pandemic threats. Participants in a previous qualitative study of eight modelers and public health practitioners noted that effective use of models during the 2009 influenza pandemic suffered because partnerships between modelers and practitioners were not formed until the pandemic was underway^30^. Importantly, communication is a two-way street: Modelers must be more clear about the capabilities and limitations of mathematical models, as well as model interpretation; meanwhile, practitioners must better communicate the information needed from models to better protect the public from outbreaks. Without such communication and use, it is clear that models will not reach their full potential as public health tools.

## Electronic supplementary material

Supplementary Information

Dataset 1

## Data Availability

De-identified data can be found online as Supplementary Dataset 1.
