# Supplementary material for: Assessing the Use of Influenza Forecasts and Epidemiological Modeling in Public Health Decision Making in the United States
Source: Sci Rep. 2018 Aug 17;8:12406. doi: 10.1038/s41598-018-30378-w (PMC6098102; doi:10.1038/s41598-018-30378-w)
Supplement: Supplementary file 1 — Supplementary Information [file 41598_2018_30378_MOESM1_ESM.pdf]

Supplementary Information

Assessing the Use of Influenza Forecasts and Epidemiological Modeling in Public Health Decision Making: Results from a Cross-Sectional Survey

Colin Doms, MA; Sarah C. Kramer, BS; and Jeffrey Shaman, PhD

Columbia University Pilot Project Survey

Page 1

Thank you for participating in our survey. We are interested in learning more about the use of influenza forecasts in public health decision making. This survey should only take a few minutes to complete, and your answers will help us better understand the use of forecasts. Your responses are confidential. In addition, this survey is voluntary. If you have questions about the survey, please email cfd2113@columbia.edu. Thank you for helping with this study.

Page 2

Influenza forecasts are predictions of future influenza incidence and are generated during the influenza season. They provide predictions of the epidemiological progression of a local outbreak, including local number of weekly cases, outbreak duration, and the week when influenza incidence peaks.

1. Are you aware that influenza forecasts are currently available?

    - Yes
    - No

Page 3

2. Have you or any colleagues seen a flu forecast in the last 12 months?

    - Yes
    - No

Page 4

3. What sources do you use to get influenza incidence or forecast information? Please select all that apply.

- CDC
- Google Flu Trends
- Columbia University
- HealthMap FluCast
- I don't use any of these sources listed
- Other (please specify)

Page 5

4. Have you or your colleagues accessed the influenza forecasts from Columbia University at http://cpid.iri.columbia.edu/flu.html?

    - Yes (sends to Page 6)
    - No (sends to Page 9)

Page 6

5. How have you used the influenza forecasts in decision making at work? Please select all that apply.

    - Reallocated money.
    - Changed communication to public or key stakeholders.
    - Affected preparedness in healthcare facilities.
    - Spurred new research.
    - I have not used them in decision making.
    - Other (please specify)

Page 7

6. The influenza forecasts from Columbia University are trustworthy.

    - Strongly disagree
    - Disagree
    - Neither disagree nor agree
    - Agree
    - Strongly agree

Page 8

7. The influenza forecasts from Columbia University are released

- Very rarely
- Somewhat rarely
- Neither rarely nor frequently
- Somewhat frequently
- Very frequently

Page 9

8. Do you use epidemiological models in your work, such as, but not limited to, infectious disease forecasts or quantitative estimates of diseases or conditions?

    - Yes (sends to Page 10)
    - No (sends to Page 12)

Page 10

9. How valuable is influenza and epidemiological modeling in your work?

    - Not at all
    - A little bit
    - Some
    - Quite a bit
    - A tremendous amount

Page 11

10. How often do you use these models?

    - Very rarely
    - Somewhat rarely
    - Neither rarely nor frequently
    - Somewhat frequently
    - Very frequently

11. How satisfied are you with this amount of use?

    - Very dissatisfied
    - Somewhat dissatisfied
    - Neither dissatisfied nor satisfied
    - Somewhat satisfied
    - Very satisfied

Page 12

12. Do you directly communicate with those who develop and create the models?

- Yes (send to Page 13)
- No (send to Page 14)

Page 13

13. How often do you directly communicate with mathematical modelers for influenza?

- Very rarely
- Somewhat rarely
- Neither rarely nor frequently
- Somewhat frequently
- Very frequently

14. How satisfied are you with this amount of communication?

- Very dissatisfied
- Somewhat dissatisfied
- Neither dissatisfied nor satisfied
- Somewhat satisfied
- Very satisfied

Page 14

15. Communication between public health professionals and modelers could improve in which ways? Please select all that apply.

- Increased frequency of telecommunication.
- Increased frequency of face-to-face meetings.
- More plain language from modelers.
- Improved relevancy of models to public health questions.
- Other (please specify)

Page 15

16. Do you work in a public health field?

- Yes (send to Page 16)
- No (send to Page 17)

Page 16

17. In which sector do you work?

- Government
- Industry
- NGO
- Academia

Page 17

18. Do your job duties involve planning for, responding to, or dealing with seasonal influenza and its epidemiology?

- Yes
- No

Page 18

19. What is your age?

- 18-29 years old
- 30-49 years old
- 50-64 years old
- 65 years and over

20. What is your gender?

- Female
- Male

21. How many years have you worked in public health and/or epidemiology?

- 0-3 years
- 4-6 years
- 7-10 years
- 10-15 years
- >15 years

22. What is the highest level of school you have completed or the highest degree you have received?

- Less than high school degree
- High school degree or equivalent (e.g., GED)
- Some college but no degree
- Associate degree

- Bachelor degree
- Graduate degree

23. In what state or U.S. territory do you live?

- Alabama
- Alaska
- American Samoa
- Arizona
- Arkansas
- California
- Colorado
- Connecticut
- Delaware
- District of Columbia (DC)
- Florida
- Georgia
- Guam
- Hawaii
- Idaho
- Illinois
- Indiana
- Iowa
- Kansas
- Kentucky
- Louisiana
- Maine
- Maryland
- Massachusetts
- Michigan
- Minnesota
- Mississippi
- Missouri
- Montana
- Nebraska
- Nevada
- New Hampshire
- New Jersey
- New Mexico
- New York

- North Carolina
- North Dakota
- North Marianas Islands
- Ohio
- Oklahoma
- Oregon
- Pennsylvania
- Puerto Rico
- Rhode Island
- South Carolina
- South Dakota
- Tennessee
- Texas
- Utah
- Vermont
- Virginia
- Virgin Islands
- Washington
- West Virginia
- Wisconsin
- Wyoming

Page 19

24. Have you received the flu vaccine this season?

  - Yes
  - No

25. Have you had influenza in the past five years?

  - Yes
  - No

End
